# Supplementary material for: Actuation enhances patterning in human neural tube organoids
Source: Nat Commun. 2021 May 27;12:3192. doi: 10.1038/s41467-021-22952-0 (PMC8159931; doi:10.1038/s41467-021-22952-0)
Supplement: Supplementary file 1 — Supplementary Information [file 41467_2021_22952_MOESM1_ESM.pdf]

Actuation Enhances Patterning in Human Neural Tube Organoids

Abdel Rahman Abdel Fattah[1], Brian Daza[1], Gregorius Rustandi[1], Miguel Angel Berrocal-Rubio[1], Benjamin Gorissen[2], Suresh Poovathingal[3], Kristofer Davie[3], Jorge Barrasa-Fano[4], Mar Cóndor[4], Xuanye Cao[5], Derek Hadar Rosenzweig[6], Yunping Lei[5], Richard Finnell[7], Catherine Verfaillie[8], Maurilio Sampaolesi[9], Peter Dedecker[10], Hans Van Oosterwyck[4], Stein Aerts[3,10], Adrian Ranga[1*]

[1] Laboratory of Bioengineering and Morphogenesis, Biomechanics Section, Department of Mechanical Engineering, KU Leuven, Leuven, Belgium
[2] J.A. Paulson School of Engineering and Applied Sciences, Harvard University, Cambridge, MA, USA
[3] Center for Brain & Disease Research, VIB-KU Leuven, Leuven, Belgium.
[4] Biomechanics Section, Department of Mechanical Engineering, KU Leuven, Leuven, Belgium
[5] Center for Precision Environmental Health, Department of Molecular and Cellular Biology, Baylor College of Medicine, Houston, TX, USA
[6] Division of Experimental Surgery, McGill University, Montreal, Canada
[7] Center for Precision Environmental Health, Departments of Molecular and Human Genetics, Molecular and Cellular Biology and Medicine, Baylor College of Medicine, Houston, TX, USA
[8] Stem Cell and Developmental Biology, Departments of Development and Regeneration, KU Leuven, Leuven, Belgium
[9] Laboratory for Nanobiology, Biochemistry, Molecular and Structural Biology Division, Department of Chemistry, KU Leuven, Leuven, Belgium
[10] Laboratory of Computational Biology, Department of Human Genetics and VIB-KU Leuven Center for Brain & Disease Research

* email: adrian.ranga@kuleuven.be

Supplementary Figures

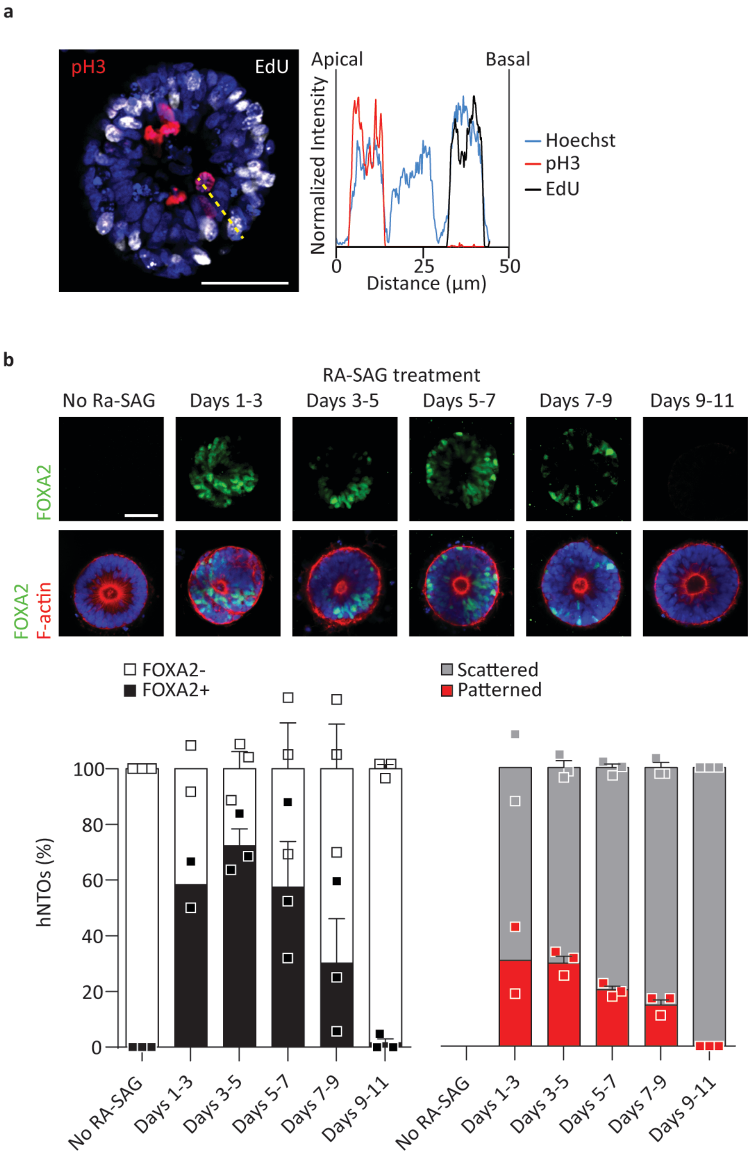

Supplementary Fig. 1 Floor plate induction and patterning modulation by RA-SAG treatment in pseudostratified epithelial hNTOs. a Representative hNTO at day 11 with mitotic cells (pH3) near the apical side and S-phase cells (EdU) near the basal side. A 50 µm line section (yellow dashed line) with normalized intensity profile of pH3 (mitotic cells, red), EdU (S-phase, black), and Hoechst (nuclei, blue) (n = 3). **b** FP induction and patterning frequency modulation upon shifting RA-SAG treatment along the experimental timeline in 2-day increments. FP expression evaluation at day 11 on fixed and permeabilized hNTOs (n = 3 except for RA-SAG treatment days 1-3 (n = 2) for > 50 hNTOs per condition). Error bars are SEM, scalebars 50 µm.

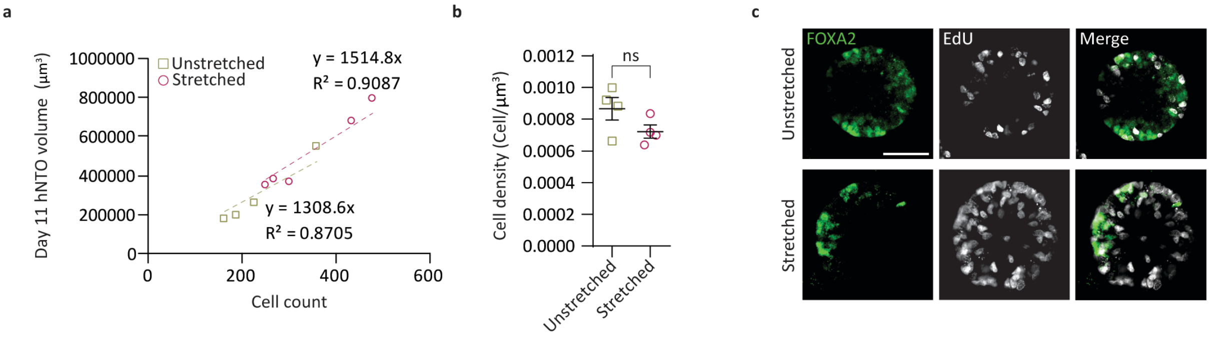

Supplementary Fig. 2 Cell count and proliferation in unstretched and stretched hNTOs. a Relationship between total cell count and organoid volume (n = 4 for unstretched hNTOs and n = 5 for stretched hNTOs). **b** Calculated cell density in unstretched and stretched organoids (n = 4 unstretched hNTOs and stretched hNTOs, Error bars are SEM, statistical analysis was determined by unpaired two-sided t-test). **c** Representative EdU stains in unstretched and stretched hNTOs demonstrating higher proliferation upon stretch (n = 2). Scalebar 50 µm.

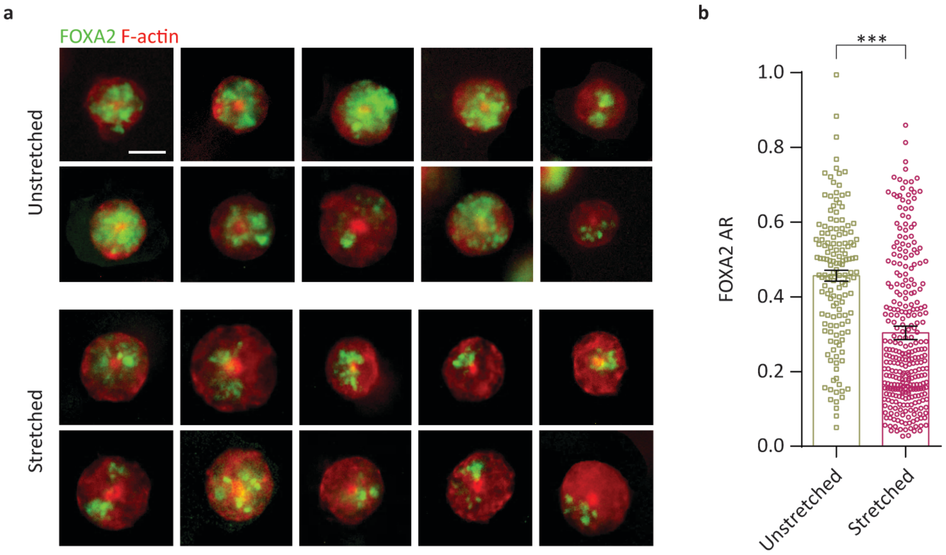

Supplementary Fig. 3 Floor plate fate in stretched and unstretched hNTOs. a Representative images of hNTOs stained for FOXA2 and F-actin in unstretched and stretched conditions. **b** FOXA2 expression in unstretched hNTOs is indicative of higher FP abundance (AR_{avg} = 0.46) compared to stretched hNTOs (AR_{avg} = 0.29) where higher patterning events are observed (data from **Fig. 1d**, statistical analysis was determined by unpaired two-sided t-test with Welch's correction $p < 0.0001$). Error bars are SEM, scalebar 50 µm.

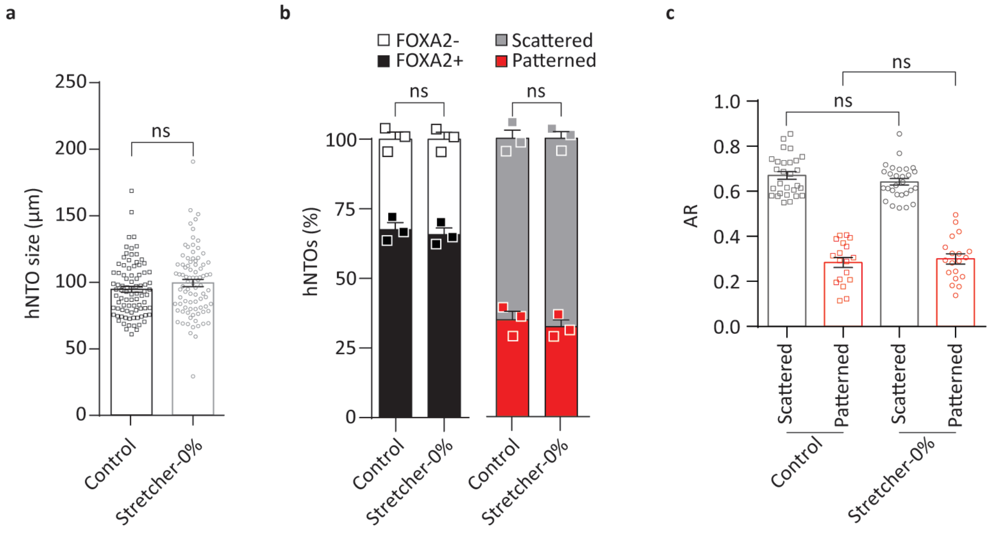

Supplementary Fig. 4 Comparison between organoids cultured in membranes in 10 cm culture dish (control) and membranes mounted on stretching devices without stretch (0% stretch). a Organoid size in control (membrane in 10 cm culture dish) and membrane in stretching device in an unstretched position (n = 3 for a total of 50 control hNTOs and 50 stretcher-0% stretch hNTOs, statistical analysis was determined by unpaired two-sided t-test). **b** FP induction and pattering frequencies for both conditions (n = 3 for a total of 68 control hNTOs and 75 stretcher-0% stretch hNTOs, statistical analysis was determined by unpaired two-sided t-test). **c** FOXA2 AR of scattered and patterned organoids for both conditions (data from **b**, statistical analysis was determined by unpaired two-sided t-test). Error bars are SEM.

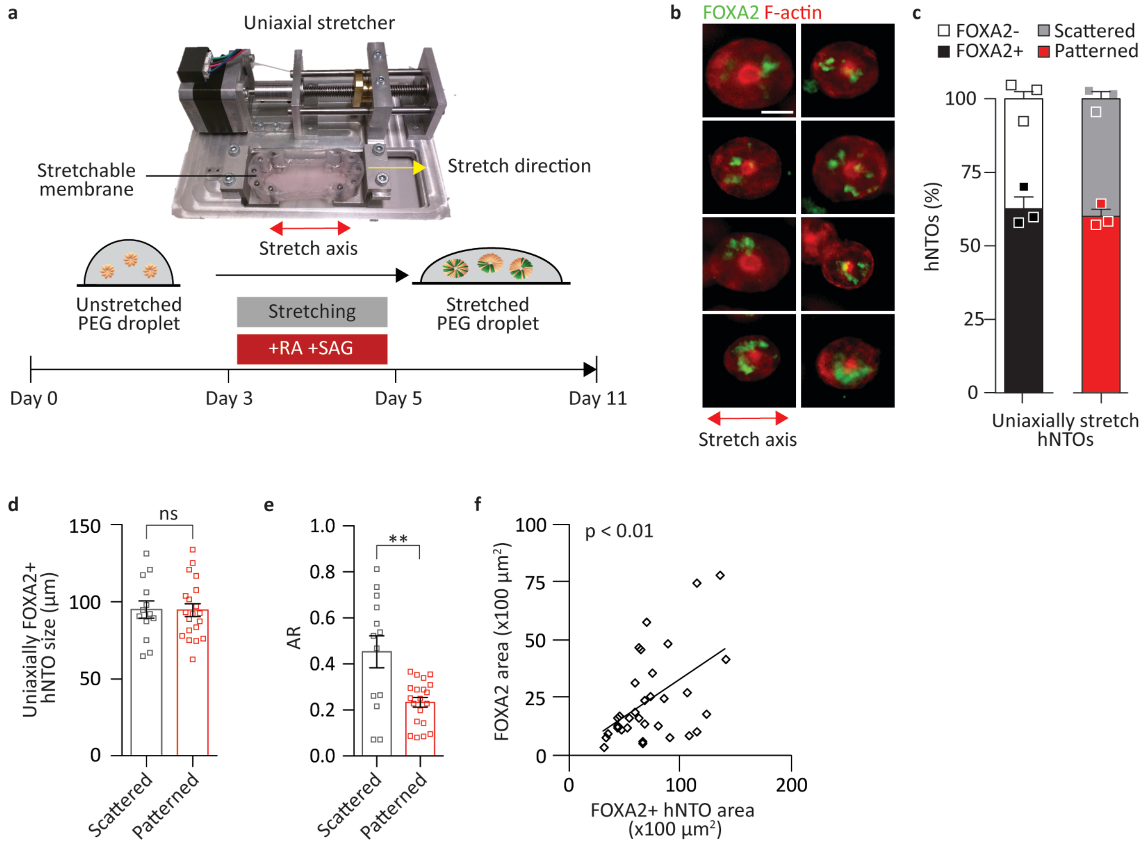

Supplementary Fig. 5 Uniaxial stretching of hNTOs. a Uniaxial stretching device and stretching/morphogen protocol. **b** Representative images displaying FOXA2 and F-actin expressions in stretched conditions (n = 3). Stretch axis is indicated by the red arrow with organoid elongation observed along the axis. **c** Quantification of FP induction and patterning for uniaxially stretched hNTOs (n = 3 for a total of 50 hNTOs). **d** Comparison of patterned and scattered organoid size in uniaxially stretched hNTOs (Pooled FOXA2+ hNTOs from **c**, statistical analysis was determined by unpaired two-sided t-test). **e** Quantification of FOXA2 area ration (AR) for scattered and patterned hNTOs upon uniaxial stretch (pooled FOXA2+ hNTOs from **c**, statistical analysis was determined by unpaired two-sided t-test with Welch's correction p = 0.0091). **f** Scatter plot of FOXA2 domain area for corresponding organoid area shows FP domain scaling with hNTO size (Pooled FOXA2+ hNTOs from **c**, p-values denote Pearson correlation analysis p = 0.0031). Error bars are SEM, ** p<0.01, scalebar 50 µm.

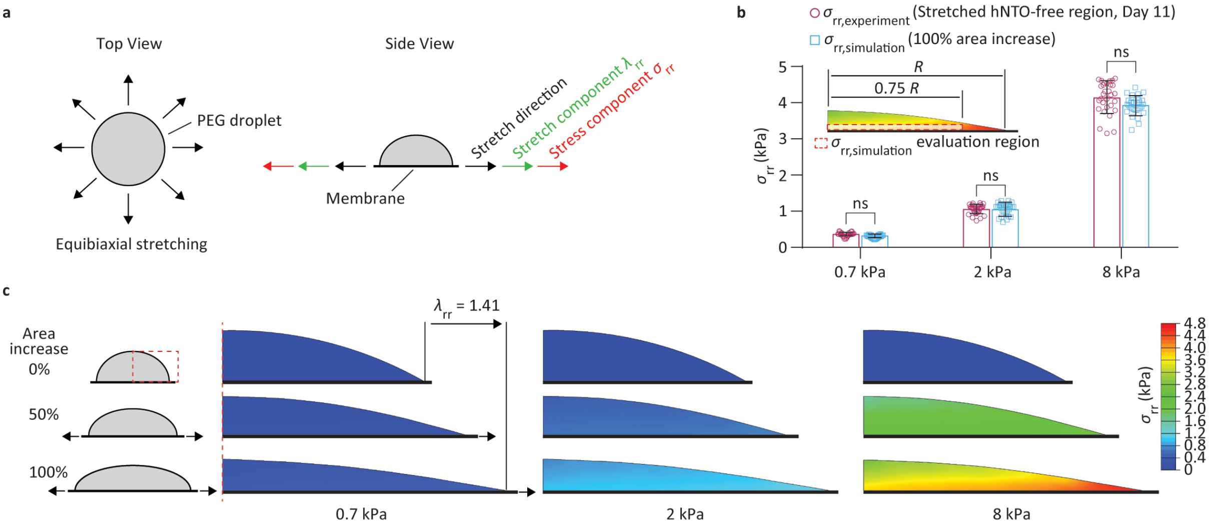

Supplementary Fig. 6 **a** Top view of equibiaxial stretching of a PEG droplet. Black arrows showing stretch direction. Side view of the same droplet showing stretch direction and associated stretch component λ_{rr} (green arrows) and stress component σ_{rr} (red arrow) in the direction of stretching. **b** Comparison between simulation stress ($\sigma_{rr,simulation}$) and experimental stress ($\sigma_{rr,experiment}$) in soft (0.7 kPa), intermediate (2 kPa) and stiff (8 kPa) matrices. $\sigma_{rr,simulation}$ evaluation at 100% area increase in the region indicated by the dashed red box over 30 equally distributed mesh elements, and $\sigma_{rr,experiment}$ evaluation in stretched hNTO-free region at day 11 (Error bars are SD, center of the error bars denote the mean, a total of 30 data points for $\sigma_{rr,simulation}$ and 30 data points for $\sigma_{rr,experiment}$ from **Fig 2a**, statistical analysis was determined by unpaired two-sided t-test). **c** Simulation results of axisymmetric equibiaxial stretching of different gel stiffnesses.

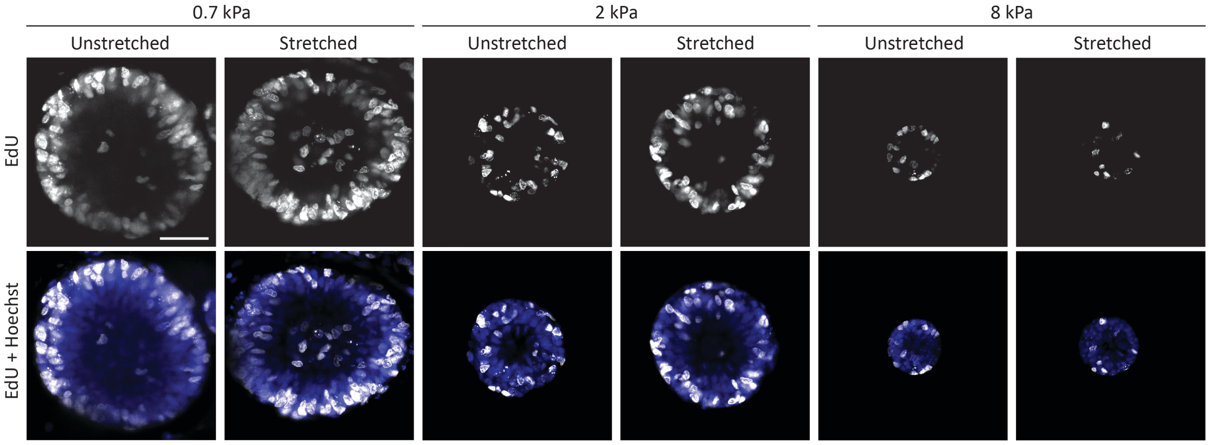

Supplementary Fig. 7 Proliferation in soft, intermediate and stiff gels. Representative EdU stains in unstretched and stretched hNTOs in soft, intermediate and stiff gel conditions. Higher EdU signal in hNTOs in soft matrices and in intermediate matrices upon stretch is indicative of increased proliferation compared to hNTOs cultured in stiff or unstretched intermediate matrices (n = 2). Scale bar 50 µm.

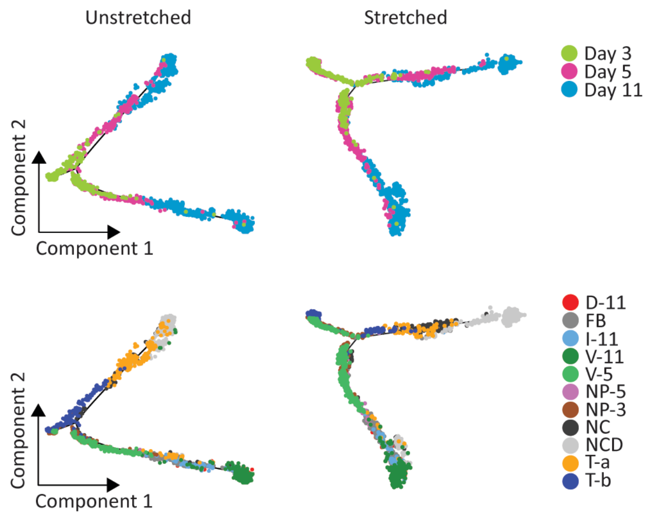

Supplementary Fig. 8 Pseudotime trajectories for unstretched and stretched hNTOs color coded for days, and annotated clusters. Trajectory progression from day 3 to day 11 with a bifurcation to 1) neural fates and 2) neural crest and neural crest derivatives fates. Comparable trajectories between unstretched and stretched hNTOs.

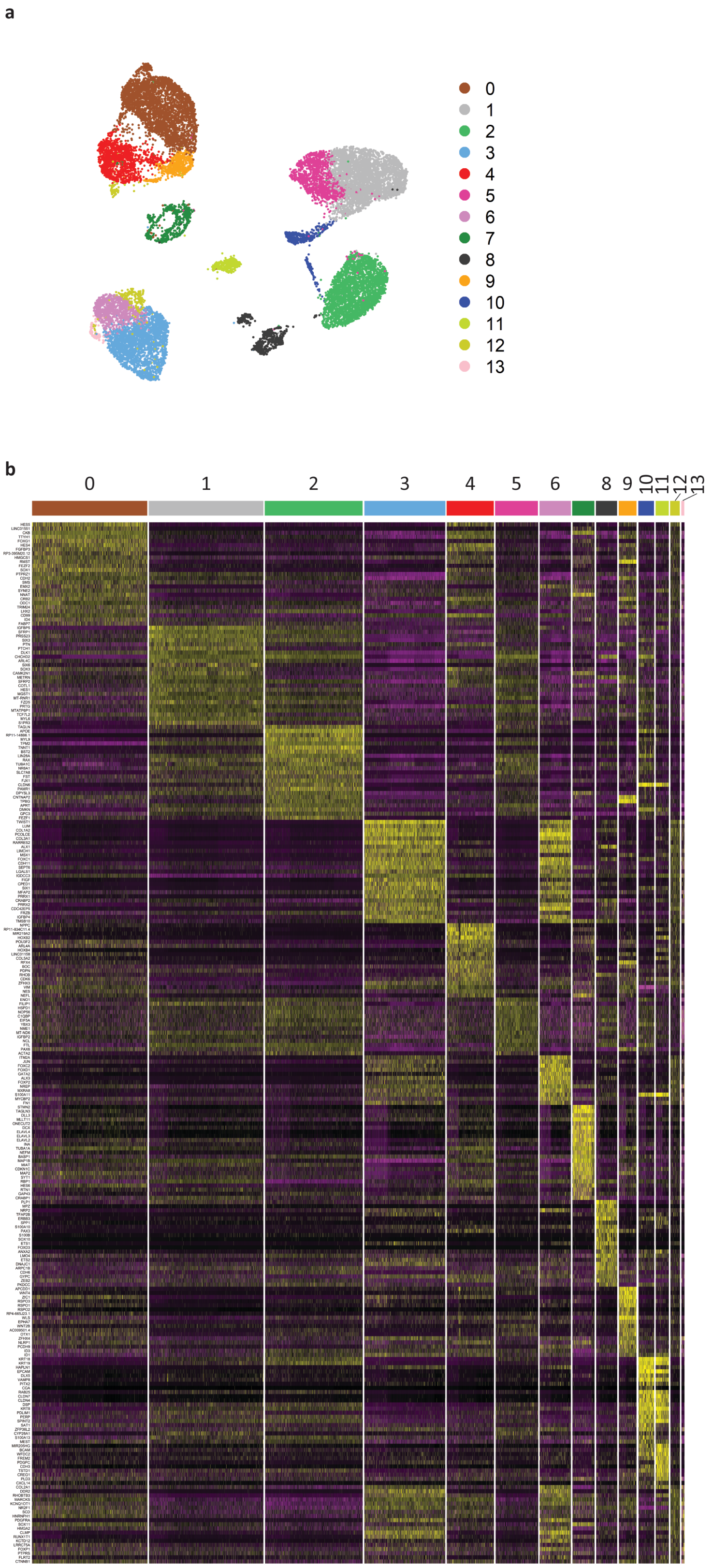

Supplementary Fig.9 UMAP and gene expression for unannotated hNTO clusters. a Unannotated UMAPs showing 14 clusters for the combined day 3, 5 and 11 unstretched and stretched dataset. **b** Gene expression heatmap showing top 25 marker genes for each cluster.

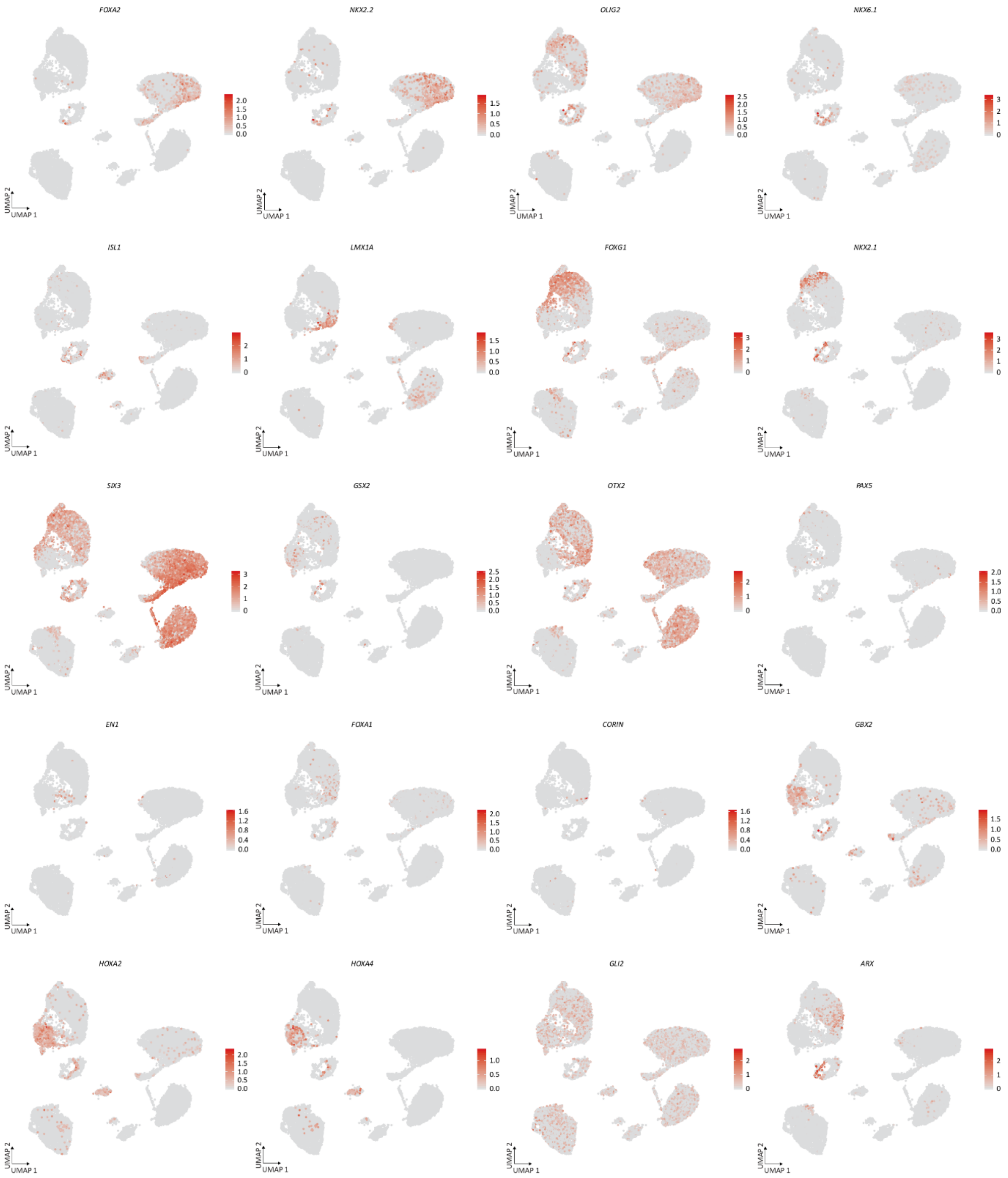

Supplementary Fig. 10 Markers of neural differentiation highlighted in UMAP representation of scRNAseq dataset.

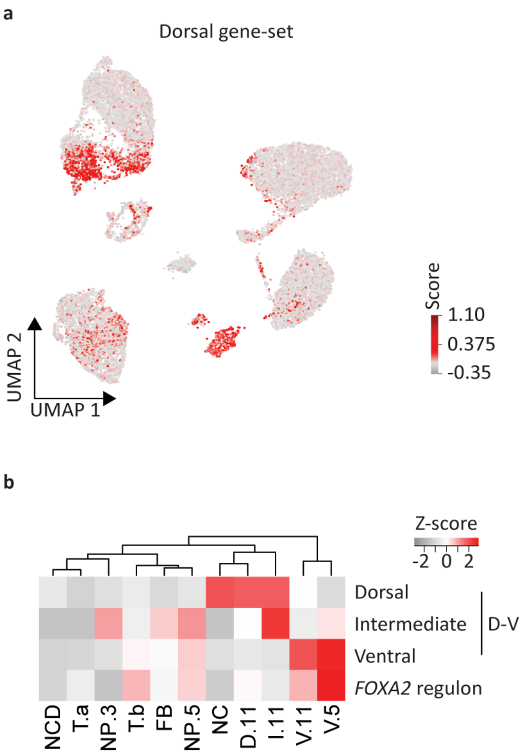

Supplementary Fig. 11 Dorsal and intermediate gene-sets and D-V hierarchical clustering. a UMAPs showing D-V (D and I, for V see **Fig. 4d**) gene-set scores. **b** Heatmap displaying hierarchically clustered clusters based on D-V gene-set scores and *FOXA2* regulon scaled AUC scores with high regulon activity correlation with the ventral identity.

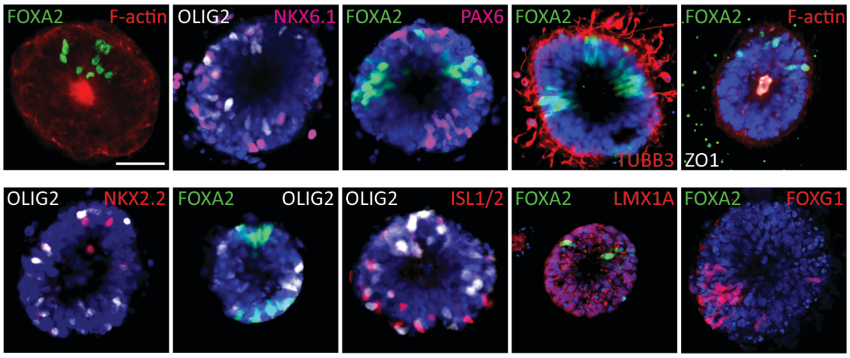

Supplementary Fig. 12 Immunohistochemistry for neural markers in stretched hNTOs. (n = 3). Scalebar 50 µm.

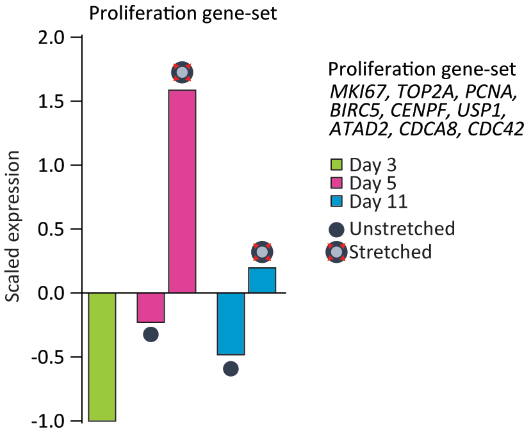

Supplementary Fig. 13 Analysis of scRNAseq dataset for gene set involved in proliferation. Scaled expression values for proliferation gene-set for day 3, 5 and 11 hNTOs in stretched and unstretched conditions.

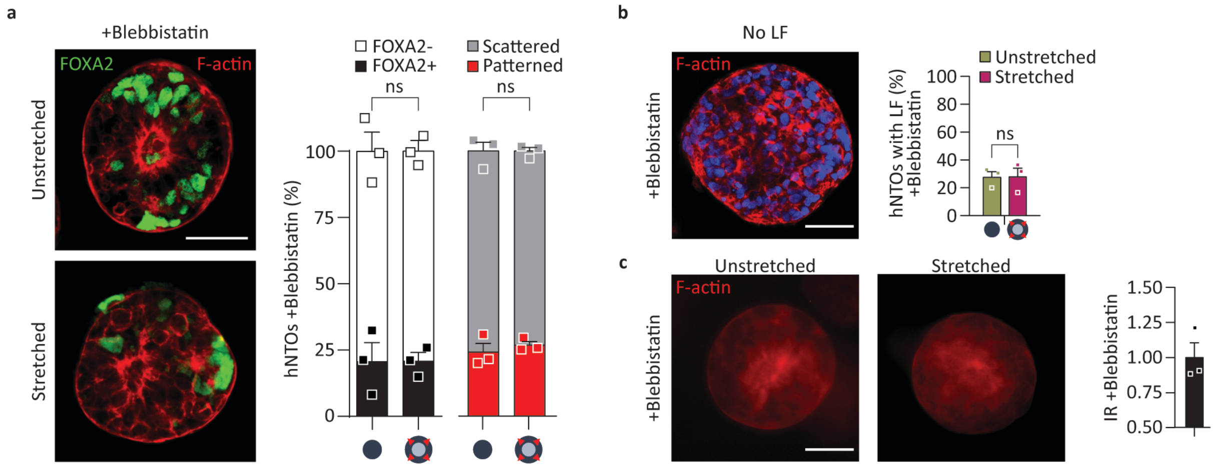

Supplementary Fig. 14 Reduction of FP induction and impaired lumen formation upon Blebbistatin treatment. **a** Representative images of FOXA2 and F-actin expressions in stretched and unstretched hNTOs exposed to Blebbistatin (10 µM, days 3-11), and quantification of FP induction and patterning in unstretched and stretched conditions (n = 3 for unstretched (436) and stretched (398) hNTOs, statistical analysis was determined by unpaired two-sided t-test). **b** Representative images showing F-actin expression for hNTOs treated with Blebbistatin (10 µM, days 3-11). Quantification of the number of hNTOs exhibiting lumen formation (LF) in stretched and unstretched conditions (n = 3 for 100 hNTO per condition, statistical analysis was determined by unpaired two-sided t-test). **c** Representative images of hNTOs treated with Blebbistatin (10 µM, days 3-11) indicating F-actin expression. Intensity ratio (IR) quantifies the relative F-actin intensity within organoids between stretched and unstretched samples (n = 3 for >25 hNTOs per condition). Error bars are SEM, scalebars 50 µm.

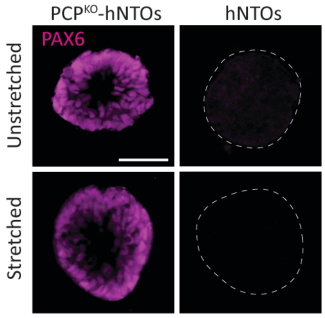

Supplementary Fig. 15 Immunohistochemistry for PAX6 in PCP^{KO}-hNTOs. Representative images displaying more abundant PAX6 expression in PCP^{KO}-hNTOs compared to control hNTOs under unstretched and stretched conditions (n = 3). Scalebar 50 µm.

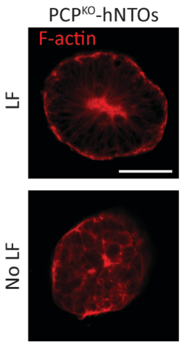

Supplementary Fig. 16 Immunohistochemistry for F-actin in PCP^{KO}-hNTOs. Disrupted cytoskeleton organization in PCP^{KO}-hNTOs resulting in elongated lumens and organoids without LF (n = 3). Scalebar 50 µm.
