# Supplementary material for: Actuation enhances patterning in human neural tube organoids
Source: Nat Commun. 2021 May 27;12:3192. doi: 10.1038/s41467-021-22952-0 (PMC8159931; doi:10.1038/s41467-021-22952-0)
Supplement: Supplementary file 2 — Reporting Summary [file 41467_2021_22952_MOESM2_ESM.pdf]

Corresponding author(s): Adrian Ranga
Last updated by author(s): Mar 1, 2021

Reporting Summary

Nature Research wishes to improve the reproducibility of the work that we publish. This form provides structure for consistency and transparency in reporting. For further information on Nature Research policies, see our Editorial Policies and the Editorial Policy Checklist.

Statistics

For all statistical analyses, confirm that the following items are present in the figure legend, table legend, main text, or Methods section.

n/a	Confirmed	
☐	☒	The exact sample size (*n*) for each experimental group/condition, given as a discrete number and unit of measurement
☐	☒	A statement on whether measurements were taken from distinct samples or whether the same sample was measured repeatedly
☐	☒	The statistical test(s) used AND whether they are one- or two-sided<br>*Only common tests should be described solely by name; describe more complex techniques in the Methods section.*
☒	☐	A description of all covariates tested
☐	☒	A description of any assumptions or corrections, such as tests of normality and adjustment for multiple comparisons
☐	☒	A full description of the statistical parameters including central tendency (e.g. means) or other basic estimates (e.g. regression coefficient) AND variation (e.g. standard deviation) or associated estimates of uncertainty (e.g. confidence intervals)
☐	☒	For null hypothesis testing, the test statistic (e.g. *F*, *t*, *r*) with confidence intervals, effect sizes, degrees of freedom and *P* value noted<br>*Give P values as exact values whenever suitable.*
☒	☐	For Bayesian analysis, information on the choice of priors and Markov chain Monte Carlo settings
☒	☐	For hierarchical and complex designs, identification of the appropriate level for tests and full reporting of outcomes
☐	☒	Estimates of effect sizes (e.g. Cohen's *d*, Pearson's *r*), indicating how they were calculated

Our web collection on statistics for biologists contains articles on many of the points above.

Software and code

Policy information about availability of computer code

Data collection	Organoid sizes, area ratio and 3D reconstruction were obtained through image evaluation on ImageJ (Version 1.52a)
Data analysis	GraphPad Prism 6 (Version 6.01), R Studio (Version 3.5.2), Seurat R Package (Version 3.0.1), R package heatmap.2, Abaqus (Dassault Systemes, 2019), MATLAB (R2018a, The MathWorks Inc.), Knee Point function (version 1.1.0.0), GOrilla web portal (http://cbl-gorilla.cs.technion.ac.il/), Revigo web portal (http://revigo.irb.hr/), Biomex (Version 1.0-0)

For manuscripts utilizing custom algorithms or software that are central to the research but not yet described in published literature, software must be made available to editors and reviewers. We strongly encourage code deposition in a community repository (e.g. GitHub). See the Nature Research guidelines for submitting code & software for further information.

Data

Policy information about availability of data

All manuscripts must include a data availability statement. This statement should provide the following information, where applicable:
- Accession codes, unique identifiers, or web links for publicly available datasets
- A list of figures that have associated raw data
- A description of any restrictions on data availability

All raw sequencing data, and the combined processed and metadata files generated in this study are available at GEO. The accession number for the reported data is (GSE154120 URL: https://www.ncbi.nlm.nih.gov/geo/query/acc.cgi?acc=GSE154120).

Field-specific reporting

Please select the one below that is the best fit for your research. If you are not sure, read the appropriate sections before making your selection.

[x] Life sciences [ ] Behavioural & social sciences [ ] Ecological, evolutionary & environmental sciences

For a reference copy of the document with all sections, see nature.com/documents/nr-reporting-summary-flat.pdf

Life sciences study design

All studies must disclose on these points even when the disclosure is negative.

Sample size	Experiments were conducted with a minimum of two independent experiments and several biological replicates (except of the scRNAseq data). To maximize sample size, the entire PEG hydrogel volume present in an experiment was analyzed for organoid fate and patterning evaluation except for the extreme edges of the hydrogel droplet. For organoid size trajectory evaluation, we relied on our previous experience to determine sample sizes that were deemed sufficient as they led to reproducible and similar results. In particular for a 95% confidence interval, a 5% margin of error and a population size of ~60 organoids (which is in the range of our obtained results) an ideal sample size is ~50 organoids. This repeatedly provided consistent and reproducible size ranges. More importantly this sample size per condition is sufficient to emphasize differences between conditions such as upon actuation, or in different hydrogel stiffness conditions.
Data exclusions	Due to slight variations in cell dissociation prior to hydrogel embedding, occasional presence of multicellular constructs (instead of single cells) can occur. These constructs are clearly identified at the beginning of the experiment and result in oversized organoids which were excluded from the data analysis. This exclusion occurred in all experiments where iPSCs were embedded in hydrogels, and the criteria was pre-established based on previous observations where initial multicellular constructs result in mishappen and irregular organoids.<br><br>Cells in the scRNAseq analysis were excluded from the analysis when their level of genes rising from the mitochondrial genome exceeded 15%. Additionally cells with fewer than 200 (low quality) and more than 7,500 (potential doublets) detected genes were also filtered out. This criteria is based on standard established protocol in the field of transcriptomic analysis and is was only applied to the scRNAseq experiment.
Replication	Data was replicated using a minimum of two and a maximum of 6 independent experiments to ensure results were reproducible. All replicates were successful except for the third replicate of the RA-SAG treatment Day 1-3 due to technical difficulties that spoiled the sample. Each condition in our scRNAseq data is the result of one replicate where day 11, day 5 and day 3 samples were performed independently.
Randomization	The starting and culture conditions were kept constant to eliminate sample-specific characteristics that can impose variations in our data, leaving the treatment (biochemical and/or mechanical) responsible for the observed variations between control and stretched samples. Within each set of replicates, samples were randomly allocated to control and different experimental groups.
Blinding	Investigators were not blinded to group allocation. The metric of patterning relied on an equation to limit investigator bias.

Reporting for specific materials, systems and methods

We require information from authors about some types of materials, experimental systems and methods used in many studies. Here, indicate whether each material, system or method listed is relevant to your study. If you are not sure if a list item applies to your research, read the appropriate section before selecting a response.

Materials & experimental systems

n/a	Involved in the study	
[ ]	[x]	Antibodies
[ ]	[x]	Eukaryotic cell lines
[x]	[ ]	Palaeontology and archaeology
[x]	[ ]	Animals and other organisms
[x]	[ ]	Human research participants
[x]	[ ]	Clinical data
[x]	[ ]	Dual use research of concern

Methods

n/a	Involved in the study	
[x]	[ ]	ChIP-seq
[x]	[ ]	Flow cytometry
[x]	[ ]	MRI-based neuroimaging

Antibodies

Antibodies used	FOXA2, Abcam (ab108422), rabbit monoclonal [EPR4466], dilution 1:200<br>FOXG1, Abcam (ab18259), rabbit polyclonal, dilution 1:200<br>pH3, Abcam (ab10543), rat monoclonal, dilution 1:200<br>TUBB3, Biolegend (802001), rabbit polyclonal, dilution 1:200<br>PAX6, Biolegend (901301), rabbit polyclonal, dilution, 1:200<br>LMX1A, Sigma (HPA030088), rabbit polyclonal, dilution 1:200

FOXA2, Santacruz (sc-374376), mouse monoclonal, dilution 1:200
OLIG2, R&D systems (AF 2418), goat polyclonal, dilution 1:200
NKX6.1, DSHB (DSHB - F55A10), mouse monoclonal, dilution 1:200
NKX2.2, DSHB (DSHB - 74.5A5), mouse monoclonal, dilution 1:200
ISL1/2, DSHB (DSHB - 39.4D5), mouse monoclonal, dilution 1:200

Alexa Fluor 555, Invitrogen (A-31570), donkey polyclonal, donkey anti mouse, dilution 1:500
Alexa Fluor 647, Invitrogen (A-31571), donkey polyclonal, donkey anti mouse, dilution 1:500
Alexa Fluor 555, Invitrogen (A-31572), donkey polyclonal, donkey anti rabbit, dilution 1:500
Alexa Fluor 647, Invitrogen (A-31573), donkey polyclonal, donkey anti rabbit, dilution 1:500
Alexa Fluor 555, Invitrogen (A-21432), donkey polyclonal, donkey anti goat, dilution 1:500
Alexa Fluor 647, Invitrogen (A-21447), donkey polyclonal, donkey anti goat, dilution 1:500
Alexa Fluor 647, Jackson ImmunoResearch (712-605-153), donkey polyclonal, donkey anti rat, dilution 1:500

Validation

Concerning antibody specificity, we kindly refer to the suppliers' websites and datasheets to find statements on specificity and relevant citations. Particularly, cellular localization of all investigated proteins have previously been reported and match the reported expressions in this study. Example publications for the primary antibodies used are mentioned below

FOXA2, Abcam (ab108422), FOXG1, Abcam (ab18259)
Jo et al., 2016, Cell Stem Cell 19:248–257 August 4, 2016

pH3, Abcam (ab10543)
Saito et al., Cell Reports 29:1555-1567.e5, 2019

TUBB3, Biolegend (802001)
Yissachar et al., 2017, Cell 168:1135–1148 March 9, 2017

PAX6, Biolegend (901301)
Quadrato et al., Nature 545(7652):48–53, May 4, 2017

LMX1A, Sigma (HPA030088)
Yamaguchi et al., Stem Cell Reports 14:1060-1075, June 9 2020

FOXA2, Santacruz (sc-374376), mouse monoclonal, dilution 1:200
Wulansari et al., Science Advances 7(8):eabb1540, February 17, 2021

OLIG2, R&D systems (AF 2418), NKX6.1, DSHB (DSHB - F55A10) and ISL1/2, DSHB (DSHB - 39.4D5)
Ranga et al., PNAS 113(44):E6831-E6839, October 14, 2016

NKX2.2, DSHB (DSHB - 74.5A5)
Luisier et al., Nature Communications 9:2010, May 22, 2018

Eukaryotic cell lines

Policy information about cell lines

Cell line source(s)

ZO1 hIPS cell line (Mono-allelic mEGFP-Tagged TJP1 WTC hPSC Line, Coriell institute for Medical Research)
A hIPS cell line reported in Sahakyan, V. et al. Sci Rep 8, 018-21103 (2018) - cell line obtained from the Stem Cell Institute Leuven (SCIL)
NCRM-1 (RRID:CVCL_1E71) hIPSC line from NIH Center for Regenerative Medicine (CRM); Bethesda; USA.

Authentication

All hIPS cell line have been authenticated by the original sources or internally by immunostaining for pluripotency markers.

Mycoplasma contamination

All cell lines tested negative for mycoplasma contamination

Commonly misidentified lines
(See ICLAC register)

No commonly misidentified cell lines listed by ICLAC were used in this work
