# Supplementary material for: Actuation enhances patterning in human neural tube organoids
Source: Nat Commun. 2021 May 27;12:3192. doi: 10.1038/s41467-021-22952-0 (PMC8159931; doi:10.1038/s41467-021-22952-0)
Supplement: Supplementary file 3 — Description of Additional Supplementary Files [file 41467_2021_22952_MOESM3_ESM.pdf]

Description of Additional Supplementary Files

File Name: Supplementary Data 1
Description: Top 25 marker genes per annotated cluster

File Name: Supplementary Data 2
Description: Regulon AUC score per annotated cluster

File Name: Supplementary Data 3
Description: List of primary antibodies
